# Extractable Free Polymer Chains Enhance Actuation Performance of Crystallizable Poly(ε-caprolactone) Networks and Enable Self-Healing

**DOI:** 10.3390/polym10030255

**Published:** 2018-03-01

**Authors:** Muhammad Farhan, Tobias Rudolph, Ulrich Nöchel, Karl Kratz, Andreas Lendlein

**Affiliations:** 1 Institute of Biomaterial Science and Berlin-Brandenburg Center for Regenerative Therapies (BCRT), Helmholtz-Zentrum Geesthacht, Kantstr. 55, 14513 Teltow, Germany; muhammad.farhan@hzg.de (M.F.); tobias.rudolph@hzg.de (T.R.); ulrich.noechel@hzg.de (U.N.); karl.kratz@hzg.de (K.K.); 2 Institute of Chemistry, University of Potsdam, 14476 Potsdam-Golm, Germany

**Keywords:** shape-memory polymer actuators, soft actuators, self-healing, poly(ε-caprolactone), thermoplastics

## Abstract

Crosslinking of thermoplastics is a versatile method to create crystallizable polymer networks, which are of high interest for shape-memory actuators. Here, crosslinked poly(ε-caprolactone) thermosets (cPCLs) were prepared from linear starting material, whereby the amount of extractable polymer was varied. Fractions of 5–60 wt % of non-crosslinked polymer chains, which freely interpenetrate the crosslinked network, were achieved leading to differences in the resulting phase of the bulk material. This can be described as “sponge-like” with open or closed compartments depending on the amount of interpenetrating polymer. The crosslinking density and the average network chain length remained in a similar range for all network structures, while the theoretical accessible volume for reptation of the free polymer content is affected. This feature could influence or introduce new functions into the material created by thermomechanical treatment. The effect of interpenetrating PCL in cPCLs on the reversible actuation was analyzed by cyclic, uniaxial tensile tests. Here, high reversible strains of up to ∆ε = 24% showed the enhanced actuation performance of networks with a non-crosslinked PCL content of 30 wt % resulting from the crystal formation in the phase of the non-crosslinked PCL and co-crystallization with network structures. Additional functionalities are reprogrammability and self-healing capabilities for networks with high contents of extractable polymer enabling reusability and providing durable actuator materials.

## 1. Introduction

Bulk materials consisting of linear polymers are controlled by the chemical structure as well as the capability to exhibit physical interactions of the macromolecules, and can be further influenced by chemical intermolecular crosslinks leading to hindered mobility of the chains. Observations for crosslinked polymeric materials can be divided into three different levels: (i) the macroscopic stability of the structure; (ii) phase morphology; and (iii) the molecular polymer network architecture. At the first level, crosslinking of thermoplastic polymer chains leads to enhanced thermomechanical stability in the thermoset in comparison to the non-crosslinked polymer, which will improve the macroscopic form stability. The stability of multiphase polymer networks (e.g., semi-crystalline systems) is controlled by phase morphologies determining the interaction of different subunits e.g., mobile or rigid content, under loading conditions. Functional moieties (e.g., polymerizable end groups) in the polymer chains allow selectively addressing and reactions forming crosslinks between different polymer chains required for the formation of network architectures [1,2,3,4]. In perfect chemical networks, the macromolecular level controls the physical properties, which are influenced by the crosslinking density, the average chain length between two netpoints, and the nature of the repeating unit. Under mechanical strain the stability of these networks is much higher, but also lead to rigid materials with a low loading capacity. Interpenetrating polymer networks (IPNs), in which two individual regular networks are combined into one interpenetrated network, show improved stability under loading as the two networks can be chosen by design according to the requirements of the system [5,6,7]. An interesting subclass of IPNs are semi-IPNs, which are composed of one crosslinked network and a linear interpenetrating polymer fraction. Here, the crosslinkable content is chemically linked, while the linear fraction is interpenetrating and introducing new features to the resulting material. Such materials can be obtained: (i) by the combination of inert polymer chains (linear content) and reactive monomers, which will form the network as soon as the reaction mixture is initiated and the polymerization proceeds; or (ii) by random crosslinking of non-functionalized linear polymers in the bulk (in case of semi-crystalline polymers in the amorphous parts). Random crosslinking by irradiation with ionizing radiation (e.g., γ irradiation), UV light, or thermal treatment in the presence of crosslinking agents or even without, enable variations in the degree of crosslinking and network formation [8,9,10,11,12,13,14,15,16,17,18]. While the macroscopic level for semi-IPNs remains similar to perfect IPNs, the network morphology is influenced depending on the crosslinking efficiency as the network might not be regular anymore. Random crosslinking might be homogenous over the sample but on the macromolecular level inter- and intramolecular crosslinks are expected, which leads to e.g., free/non-bonded chains, or dangling chains, and changes the average chain length between two netpoints. Controlling the degree of crosslinking and therefore the amount of crosslinked chains, which contribute “actively” to the formation of a network, allows to influence the physical properties of the resulting networks [10].

Beside these properties, new functions after thermal or thermo-mechanical treatment can be achieved through the presence of linear polymer, which enables the formation of multifunctional networks. For example in bulk materials, increased energy dissipation mechanisms upon deformation of samples are observed, as the linear content can move spontaneously within the bulk material without destruction of chemical links, enabling sliding and strain hardening of macromolecules [19,20]. For shape-memory polymer actuators, crosslinks play an important role as the formed netpoints retain the permanent shape of the materials as it needs to be heated. On the other hand, the network helps to stabilize the orientation of crystallizable chain segments in such systems [8,21,22]. In so-called “one-way” and “two-way” shape-memory polymer actuators, which can act under stress-free conditions and under stress [21,23,24,25,26,27], linear fractions could influence the performance of actuation between different shapes due to differences in the crystallization behavior of linear and restricted crosslinked polymer chains. Furthermore, linear chains of moderate molecular weight typically show low recovery, based on entropic recovery induced by entanglements, but high plastic deformation, while covalent networks show more elastic recovery, after applying strain, which will influence the overall behavior. The understanding of such network relationships would allow us to design new multifunctional actuator materials according to the requirements and the environment. Here, especially insights into the phase morphology should play a key role as this controls the interaction between the macroscopic level and the molecular level. Influencing this regime might allow us to tune/manipulate the properties resulting from the material, by varying the amount of extractable polymer chains in the network, in the context of bidirectional shape-memory actuation and self-healing capability.

As a suitable material candidate, poly(ε-caprolactone) (PCL) is crosslinked by different techniques, like irradiation with ionizing radiation (e.g., γ irradiation), UV light, and thermal treatment, leading to different amounts of extractable fractions. PCL was chosen due to its great versatility in blends, fibers, or networks being able to undergo shape-memory or even reversible shape-memory effects [8,11,28,29,30]. Non-crosslinked PCL fractions of 5–60 wt % were achieved and network composites were explored concerning their crosslinking density, physical properties, and distribution of the non-crosslinked polymer chains in the material. These findings can be translated into contributions on the reversible bidirectional shape-memory capability, which is characterized by cyclic, thermomechanical uniaxial tensile tests under stress-free conditions. Therefore, the function of the linear content on the mechanical properties, the morphology in the material, and the influence on the shape-memory actuation were compared for the different compositions. Additional functions like reprogrammability and self-healing induced by the linear fraction were addressed and studied in this work, as it allows the formation of reusable actuation materials.

## 2. Materials and Methods

### 2.1. Materials

Semi-crystalline poly(ε-caprolactone) (PCL) with a number average molecular weight of 41,000 g∙mol^−1^ (CAPA^®^ 6800, Solvay, Germany) was used for this study. Dicumyl peroxide (DCP 98%) and triallyl isocyanurate (TAIC 99%) served as thermal and photo initiator, respectively, while benzophenone (BP ≥ 99%) was employed as a crosslinker for UV irradiated networks. Chloroform (≥99.8%) was used to purify the crosslinked networks and to determine the degree of gelation (*G*), whereas dioxane (99.8%) was used for swelling and freeze drying of extracted networks for morphological investigations. All these reagents were purchased from Sigma-Aldrich (Steinheim, Germany) and used without any purification. 

Network Preparation: the networks with different amounts of non-crosslinked PCL were prepared according to the respective synthesis protocol below. The resulting materials are named cPCL*XX*-L*YY* depending on the composition indicated by numbers respective to the weight fraction of crosslinked (cPCL) or of non-crosslinked/linear polymer (L).

cPCL*95*-L*05*: networks were prepared by mixing 98 g of PCL and 2 g solution (50:50) of BP and TAIC in an extruder followed by film formation using compression molding. These films were subsequently irradiated by a UV curing system (model I300MB with an electrodeless mercury lamp, 300 watt inch^−1^, Fusion UV systems GmbH, Ismaning, Germany) for 3 min at 50 °C. 

cPCL*83*-L*17*: networks were prepared by mixing 99 g of PCL and 1 g TAIC in extruder and the blends after compression molding into films were cross-linked by electron beam irradiation of 99 kGy intensity.

cPCL*70*-L*30*: was prepared by mixing 98 g of PCL and 2 g DCP in a twin-screw extruder (Euro Prism Lab, Thermo Fisher Scientific, Waltham, MA, USA) at 110 °C and 50 rpm. The polymer/DCP blends were compression molded into 2D films on a compression molding machine (type 200 E, Dr. Collin, Ebersberg, Germany) with 1 mm thickness and subsequently crosslinked at 200 °C and 120 bar for 25 min. 

cPCL*40*-L*60*: networks were prepared by irradiating a film of linear PCL without any initiator or cross-linker inside via an electron beam dose of 165 kGy.

cPCL*XX:* networks free of extractable polymer chains were obtained by intensive extraction with chloroform until no further weight loss was determined after drying.

### 2.2. Methods

Degree of Gelation and Degree of Swelling: Crosslinked polymer networks were analyzed by swelling experiments in chloroform at ambient temperature, extraction time 24 h, with another 24 h for solvent evaporation in a vacuum oven under reduced pressure. To ensure the complete removal of linear polymer, this process was repeated three times. The gelation degree *G* was calculated from the isolated weight *m*_iso_ of the sample and the dry weight *m*_d_ after extraction using Equation (1) [31]

(1) $$G=\frac{m_{d}}{m_{\mathrm{iso}}}\times 100\%$$

and the swelling degree *Q* was calculated by Equation (2):

(2) $$Q=1+\rho_{2}\cdot \left(\frac{m_{\mathrm{sw}}}{m_{d}\cdot \rho_{1}}-\frac{1}{\rho_{1}}\right)\times 100\%$$

where *m*_sw_ is the weight of the sample in the swollen state, *m*_d_ is the dry weight of the extracted sample, and *ρ*_1_ and *ρ*_2_ are the specific densities of the swelling medium and the polymer, respectively. Density of PCL was 1.14 g∙cm^−3^ and for chloroform, 1.483 g∙cm^−3^ was used (supplier data).

Differential Scanning Calorimetry (DSC) measurements were performed on a calorimeter (Netzsch, Selb, Germany) DSC 204, with heating and cooling rates of 10 K∙min^−1^. Thermal data reported correspond to the second heating, whereas the area under the melting and crystallization peaks led to calculation of the corresponding enthalpies. Weight % crystallinity was calculated according to Equation (3) [32].

(3) $$\chi_{c}=\frac{\Delta H_{m}}{\Delta H{{}^{\circ}}_{m/100}}\times 100\%$$

where ∆*H°*_m/100_ is enthalpy of melting of 100% crystalline polymer, which is 134.9 J∙g^−1^ for PCL [22].

Scanning Electron Microscope (SEM): Extracted and non-extracted cPCL samples were examined by SEM (Phenom™, L.O.T.-Oriel GmbH & Co., KG, Darmstadt, Germany) to determine structural changes after extraction. For the removal of the non-crosslinked PCL specimens were swollen in chloroform as described in the previous section. Surface and cross-sections were analysed, whereas the cross-sections were prepared by freeze fracture method in liquid nitrogen. Samples were sputtered (5 nm gold layer) and images with 2700×–1300× magnifications were obtained. Similarly, freeze-dried cPCLs obtained from dioxane were analysed by SEM.

Tensile tests at ambient temperature were performed (Z1.0, Zwick, Ulm, Germany) by elongation of samples until break, while tensile tester Zwick Z1.0 (Zwick, Ulm, Germany) equipped with a thermo-chamber and temperature controller (Eurotherm Regler, Limburg, Germany) was used for tensile tests above *T*_m_ = 90 °C. 

Bending tests: Tensile bar-shaped samples were programmed by bending them to 180° in a complete amorphous state in hot water (*T*_prog_ ≈ 90 °C) and subsequent quenching in ice cold water (≈5 °C) to give a hairpin like shape. Angle change was realized by subsequent heating in hot water (*T*_high_ ≈ 50 °C) and cooling in cold water (*T*_low_ ≈ 5 °C). Quantification of reversible actuation in bending experiments count in a reversible change in angle (θ´) as

(4) $$\theta^{\prime }=\theta \left(T_{\mathrm{high}}\right)-\theta \left(T_{\mathrm{low}}\right)$$

where θ(*T*_high_) is the angle of the test specimen observed in hot water and θ, θ(*T*_low_) is the angle observed in ice cold water.

Determination of network properties: Tensile tests at 90 °C were analyzed by the Mooney-Rivlin approach (plot between reduced stress and reciprocal stretch ratio), where the crosslinking density (*υ*_c_) and molecular weight (*M*_c_) of the network chain segments between crosslink points were calculated by using Equations (5) and (6) respectively [33].

(5) $$\upsilon_{c}=\frac{\left(2C_{1}+2C_{2}\right)}{RT}$$

(6) $$M_{c}=\frac{\rho_{2}RT}{2C_{1}}$$

Here, *C*_1_ and *C*_2_ are the Mooney-Rivlin constants, *R* is the gas constant, *T* is the temperature at which tensile tests were performed and *ρ*_2_ is the specific density of the polymer. The constant *C*_1_ is related to the number of covalent net points in the crosslinked network, whereas *C*_2_ is associated with the contribution from trapped entanglements including physical crosslinks of the network.

Crosslinking density (*υ*_c_) and average molecular weight between two neighboring netpoints (*M*_c_) were calculated by means of Flory-Rehner Equation (7) using swelling experiments. Here *φ*_2_ is the polymer volume fraction in the swollen system, which is experimentally determined as the ratio of volume of the dry sample (*V*_d_) to that of swollen sample (*V*_sw_) as φ_2_ = *V*_d_/*V*_sw_, while *V*_1_ is the molar volume of the solvent. Parameters for chloroform were taken from the supplier: density 1.48 g·cm^−3^ and molar mass 119.38 g·mol^−1^, and molar volume 80.662 cm^3^·mol^−1^.

(7) $$\upsilon_{c}=\frac{\ln\left(1-\varphi_{2}\right)+\varphi_{2}+\varphi_{2}^{2}\chi_{12}}{V_{1}\left[\frac{\varphi_{2}}{2}-\varphi_{2}^{\frac{1}{3}}\right]}$$

Flory solvent-polymer interaction parameter (χ) for chloroform and PCL at room temperature was taken from the literature as χ = 0.045 ± 0.004 [34].

Cyclic, thermomechanical tests were carried out on a Zwick Z1.0 with a thermo-chamber as described for tensile tests above *T*_m_. The cyclic experiments consisted of an initial programming cycle and three reversible actuation cycles. During programming, the sample was stretched to ε_prog_ (60% of the maximum ε_b_) at *T*_prog_ = 90 °C followed by equilibration time of 10 min and was cooled to 0 °C under constant strain. After another 10 min equilibration time, the stress was released to zero stress at this lower temperature and the sample was reheated to *T*_high_ under stress-free conditions. The actuation cycle consisted of cooling to *T*_low_ = 10 °C, waiting for 10 min and reheating to *T*_high_ = 60 °C followed by another 10 min waiting time. The heating and cooling rates were 2 K∙min^−1^ in cyclic experiments and the strain rate was 5 mm∙min^−1^ in all the mechanical and cyclic experiments. Quantification of the reversible actuation includes switching temperatures for actuation and recovery as well as the reversible change in strain (Δε) calculated as the difference of the strain at *T*_low_ and *T*_high_.

In situ small-angle X-ray Scattering (SAXS) measurements were performed on a Nanostar diffractometer (Bruker AXS, Karlsruhe, Germany) using a two-dimensional VANTEC-2000 detector and X-rays of 0.154 nm wavelength, whereas the distance of the sample from detector was 1070 mm calibrated with silver behenate standard. A tree-pinhole collimation system gave a beam size of 400 µm. Samples were initially programmed by deforming to ε_prog_ = 400% at *T*_prog_ = 90 °C, followed by cooling to 0 °C and two reversibility cycles with *T*_high_ = 60 °C and *T*_low_ = 10 °C at constant heating/cooling rates of 2 K∙min^−1^. The scattering patterns were integrated after background subtraction over a 10° wide chi range along the *s*_3_ axis (deformation direction) where discrete peaks were observed, leading into a one-dimensional curve *I* vs. *s*_3_. Long periods were determined from the position of the peak maxima after Lorentz correction (*I*(s) → s^2^*I*(s)) as *L* = 1/*s*_max_.

Wide-angle X-ray Scattering (WAXS) measurements on programmed samples (as for SAXS) were performed using a D8 Discover diffractometer with a two-dimensional Hi-Star-detector (105-μm pixel size; Bruker AXS, Karlsruhe, Germany) using X-rays with 0.154 nm wavelength. A three-pinhole collimator with an opening of 0.8 mm was used. The distance between sample and detector was 150 mm, whereas the samples with a thickness of about 1 mm were illuminated for 2 min in transmission geometry. In situ measurements at fixed stages during programming were performed using a custom-build stretching device, a heating gun, and a cooled nitrogen gas stream at 5 min exposure time per scattering pattern.

Self-healing capabilities from damaged dumb-belled shaped specimens (width/length 2 mm × 30 mm) were estimated, where a centered cut of approximately width/length 250 µm × 300 µm was made by laser cutting on each side. Healing was done above the melting transitions (˃70 °C) and healing efficiency was calculated as described in Equation (8),

(8) $$\text{Healing efficiency}=\frac{\varepsilon_{b,\mathrm{healed}}}{\varepsilon_{b,\mathrm{virgin}}}\times 100$$

where ε_b,healed_ is elongation at break of the healed sample, while ε_b,virgin_ is the elongation at break of non-damaged fresh sample.

## 3. Results

Bulk films consisting of poly(ε-caprolactone) are crosslinked, leading to polymer networks with different extents of crosslinked PCL (cPCL) due to the varying crosslinking approaches (details see Materials and Methods). All methods should lead to homogenous networks throughout the material, which turn the thermoplastic material into form-stable thermosets. The fraction of extractable polymer chains was determined by calculating the gel content after intensive extraction. Via this approach materials containing 60–5 wt % extractable contents were obtained. The set of polymer networks was named according to their compositions, cPCL*95*-L*05*, cPCL*83*-L*17,* cPCL*70*-L*30*, and cPCL*40*-L*60*, respectively, where the numbers indicate the weight fraction of the network (cPCL) and non-crosslinked/linear content (L). Via these different compositions we studied and compared the changes in the microstructural architecture of the network, the influence of the linear content on the actuation capability, and self-healing induced by the mobility of such non-crosslinked PCL chains.

**Figure 1:**
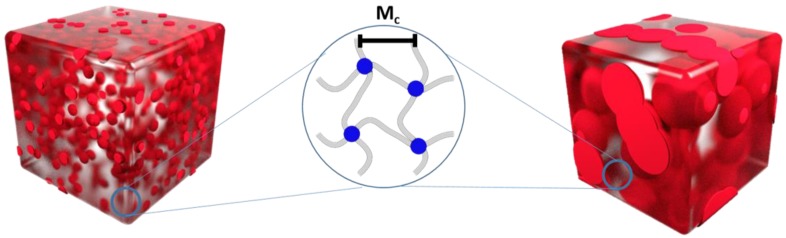
Schematic representation of networks containing different amounts of non-crosslinked PCL leading to different morphologies containing open or closed compartments defining the accessible reptation volume for the macromolecules (red domains). Network structure on the macromolecular level indicating a similar average chain length (M_c_) between netpoints.

In case of perfect polymer networks (gel content 100%), the distance between two crosslinking points is solely determined by the molar mass of the chain connecting ends, which is also determining the size of unit cell of such a network, which can be quite small. In our system inter- and intramolecular crosslinks between PCL chains can occur within the material, which is controlled by the position of the respective crosslinking event. This controlls the crosslinking density and the amount of non-crosslinked polymer and will influence the network architecture (Figure 1). A direct visualization of the network structure and the position of free macromolecules and the network PCL is not possible. Therefore, the respective specimens were intensively extracted and on the one hand side freeze-fractured after drying, and once freeze-dried from dioxane before fracturing. As the linear content is removed from those specimens they were named as e.g., PCL*95*, where the number denotes the non-extractable network content. Scanning electron microscopy (SEM) micrographs of these samples confirmed for all extracted samples the formation of “sponge-like” structures, with closed or open compartments depending on the amount of non-crosslinked PCL (Figures S1 and S2). Samples with high contents of extractable fractions of PCL lead to a collapse of the porous structure (>30 wt %) and complete destruction of the sample (cPCL*40*; no SEM images were obtained). The existence of closed/open compartments influences the accessible volume for the reptation of linear macromolecules, hinder the chain mobility, or restrict these into confined spaces in case of closed ones. In case of open pores, an interaction between non-crosslinked polymer chains with different pores can occur.

**Figure 2:**
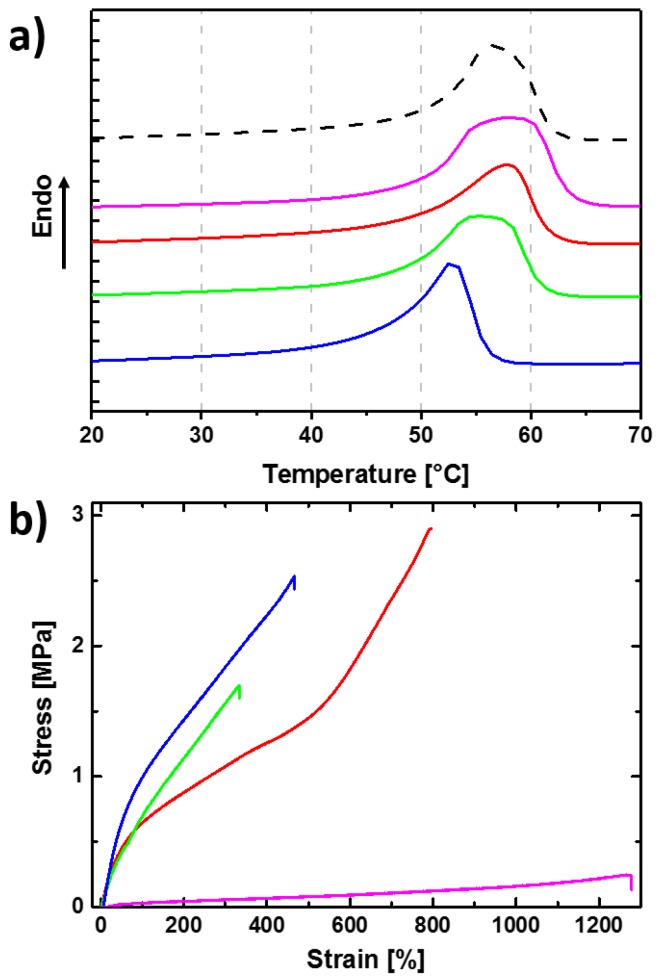
(**a**) Comparison of DSC thermograms for: cPCL*95*-L*05* (blue), cPCL*83*-L*17* (green), cPCL*70*-L*30* (red), cPCL*40*-L*60* (magenta), and linear PCL (dashed black line); (**b**) Comparison of the stress-strain curves obtained in tensile tests at 90 °C for different crosslinked PCL networks: cPCL*95*-L*05* (blue), cPCL*83*-L*17* (green), cPCL*70*-L*30* (red), and cPCL*40*-L*60* (magenta).

The thermal properties determined via differential scanning calorimetry (DSC) showed the lowest melting temperature (*T*_m_) in case of cPCL*95*-L*05* as a reduced crystal size population is obtained, which might be attributed to spatial restrictions for the non-crosslinked PCL in the compartments and the high crosslinking density reducing the chain length between two netpoints (Figure 2, Table 1). By lowering the crosslinked fraction an increase in *T*_m_ from 52 °C (cPCL*95*-L*05*) to 59 °C (cPCL*40*-L*60*) and a broadening of the melting peak is observed, correlating with the amount of free polymer chains. For the crystallization during cooling a similar trend is observed (Figure S3). The crystallinity (*χ*_c_) is not influenced and remains constant for all samples. The thermograms allowed us to separate already three important temperature ranges, which became central in the later context for testing shape-memory capabilities. At temperatures below 10 °C, all samples are in the crystalline state, at 80 °C the polymer networks are completely molten, and at 60 °C we are in the offset of all thermograms for the melting showing still some remaining crystals. 

The mechanical stability of the cPCLs was analyzed by tensile measurements at 25 and 90 °C. For the measurements at 25 °C, a clear trend for the Young’s modulus (*E*) and the elongation at break (ε_b_) correlating with the content of linear polymer chains was found (Figure S4, Table 1). *E* decreases from 260 MPa in cPCL*95*-L*05* to 175 MPa in cPCL*40*-L*60* as here the amount of free polymer increases leading to a softening of the material, while the ε_b_-values show the opposite trend from 500% to 900%, respectively, as here sliding is enhanced by increasing the amount of unbound polymer chains (Table 1, Figure S4). At elevated temperatures (90 °C) above the melting temperature of PCL a similar trend is observed in the mechanical properties, showing the thermal stability of those materials induced by the introduced crosslinks (Figure 2b). For very high contents of non-crosslinked PCL (cPCL*40*-L*60*) the contribution of linear chains to the stability hinders enhanced stability at elevated temperatures, but still leads to improvement in comparison to non-crosslinked PCL.

As the materials were formed by different crosslinking techniques and under different thermal conditions, the networks were investigated concerning their architecture, which is defined by the average chain length between two netpoints and the crosslinking density. Crosslinking of PCL itself is expected to occur between amorphous chains while the crystalline domains should not be influenced. Temperatures above and below *T*_m_ were used depending on the method, which might lead to changes in the network architecture due to the respective situation of crystal/melt. This allowed comparing general approaches for crosslinking materials described in the literature, and their corresponding influence. To get an insight into the network properties, the stress-strain curves obtained at 90 °C were analyzed by Mooney-Rivlin approach with corresponding plots between reduced stress and reciprocal stretch ratio. Mooney-Rivlin calculations enabled us to determine the crosslinking density (*υ*_c_) and the average molar mass between two netpoints (*M*_c_) (Table 2). *υ*_c_ increases with the increasing gel content for the extracted, as well as for the non-extracted specimens. Interestingly, obtained values are almost comparable for cPCL*70*-L*30*/cPCL*70* to cPCL*95*-L*05*/cPCL*95* although these materials were obtained by different crosslinking techniques. The same trend is observed as well for *M*_c_, which also only slightly decreases with a higher crosslinking density. To further confirm our finding, swelling experiments (Table S1) of the specimens were analyzed by Flory theory, which lead to a similar trend and almost similar values as for the Mooney-Rivlin determination (Table 2). With these details, we can complete the images drawn for our materials (Figure 1), which have similar average *M*_c_ and *υ*_c_ on the macromolecular level for the networks formed, no matter which approach was chosen for the synthesis, allowing us the assumption that this could be generalized to other systems as well (but this needs to be evaluated in later studies). The only parameter which is changed is the accessible volume for the non-crosslinked polymer. This should have an influence on the structure-property relationship for the different networks and the actuation capability effected by the linear content.

**Figure 3:**
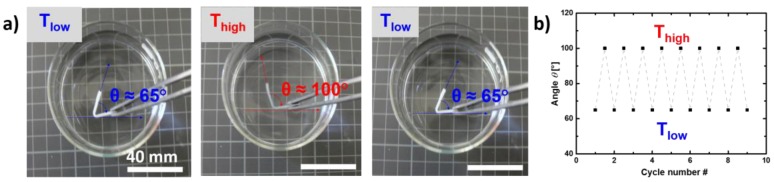
(**a**) Comparison of image series at different temperatures indicating the angle change at *T*_low_ and *T*_high_ for cPCL*95*-L*05*; (**b**) Multiple cycle experiment for the angular change between *T*_low_ and *T*_high_ for PCL*95*-L*05*.

The first function, which should be examined depending on the network composition, is the capability of the material to show stress-free shape-recovery and reversible bidirectional shape-memory effect (rbSME). The rbSME enables reversible movements of free-standing shaped bodies and differs from the two-way shape-memory effect, which is usually discussed for conditions under stress. The stress-free conditions of rbSME require a careful selection of actuation temperature ranges as only a partial melting of the domains should be achieved. Otherwise the full recovery of the sample is observed (see also reviews of the SME). For this, all networks were bended at 90 °C and the shape was fixed at 5 °C. All specimen showed shape recovery and reversible shape-memory properties except one example—cPCL*40*-L*60* (Figure S5). cPCL*40*-L*60* showed only the recovery from the bended state (programmed state) to the original shape but no macroscopic reversible effect between 5 and 50 °C. For the other samples, a reversible alteration between two states at *T*_high_ (50 °C) and *T*_low_ (5 °C) were determined by changes in the angle of the bended specimen (Figure 3a). For cPCL*95*-L*05* the high reproducibility of the reversible actuation is shown by the angular changes between 65° (at *T*_low_) and 100° (at *T*_high_) in a multiple cycle experiment (Figure 3b).

### 3.1. Reversible Actuation Capabilities

**Figure 4:**
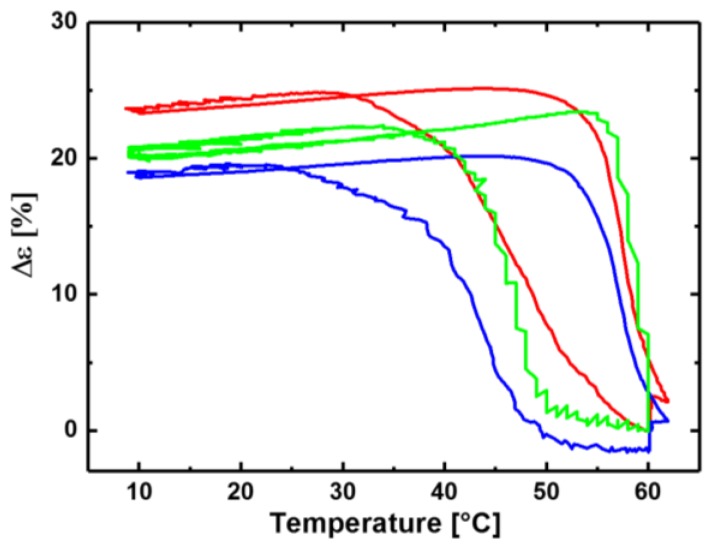
Comparison of reversible elongation of different cPCLs: cPCL*95*-L*05* (blue), cPCL*83*-L*17* (green), and cPCL*70*-L*30* (red).

For a more detailed study of the stress-free reversible actuation capability of these systems cyclic thermomechanical tensile experiments were performed. The samples are programmed at 90 °C by stretching, fixation at 0 °C, and releasing of the applied stress, followed by sequential heating/cooling cycles between 10 and 60 °C. Conceptually, actuation from shape-memory material is resulting from volume changes induced by transitions between the crystalline and molten state of crystallizable polymers. Here, the crystallization-induced elongation (CIE) and the melting-induced contraction (MIC) of the domains correlate into macroscopic changes. As for the macromolecular level the network structure is almost the same, the linear fraction should be the only parameter influencing the rbSME. cPCL*95*-L*05* shows a reduced Δε value, which determines the variation of the elongation between the different temporary states at *T*_low_ (10 °C) and *T*_high_ (60 °C), in comparison to the other sets of cPCLs. For cPCL*70*-L*30* the highest Δε of 24% is observed (Figure 4) as the linear content enhances the overall actuation capability. According to the DSC thermograms crystals with a higher thermal stability can be formed in the presence of higher amounts of non-crosslinked PCL as here we assume higher mobility of the chains and more accessible reptation volume generated in the overall structure. Interestingly, there seems to be a certain threshold, as a further increase of the linear content does not necessarily lead to additional improvements. As already observed in the bending experiment, for cPCL*40*-L*60* the determined Δε drops to 3% (Table 3), as here the network structure is too weak to retain any orientation of the PCL crystals in the material.

Actuation is always strongly connected to crystal structure and their spatial orientation in the specimen. To gain insight into the nanostructure, programmed samples of cPCL*70*-L*30* were analyzed by in situ small- and wide-angle X-ray scattering techniques (SAXS and WAXS) at different temperatures. WAXS gives information about the crystal structure, the orientation of the crystallites, and the degree of crystallinity (DOC) in the material. SAXS provides information about larger domain sizes and spatial arrangements of the domains.

**Figure 5:**
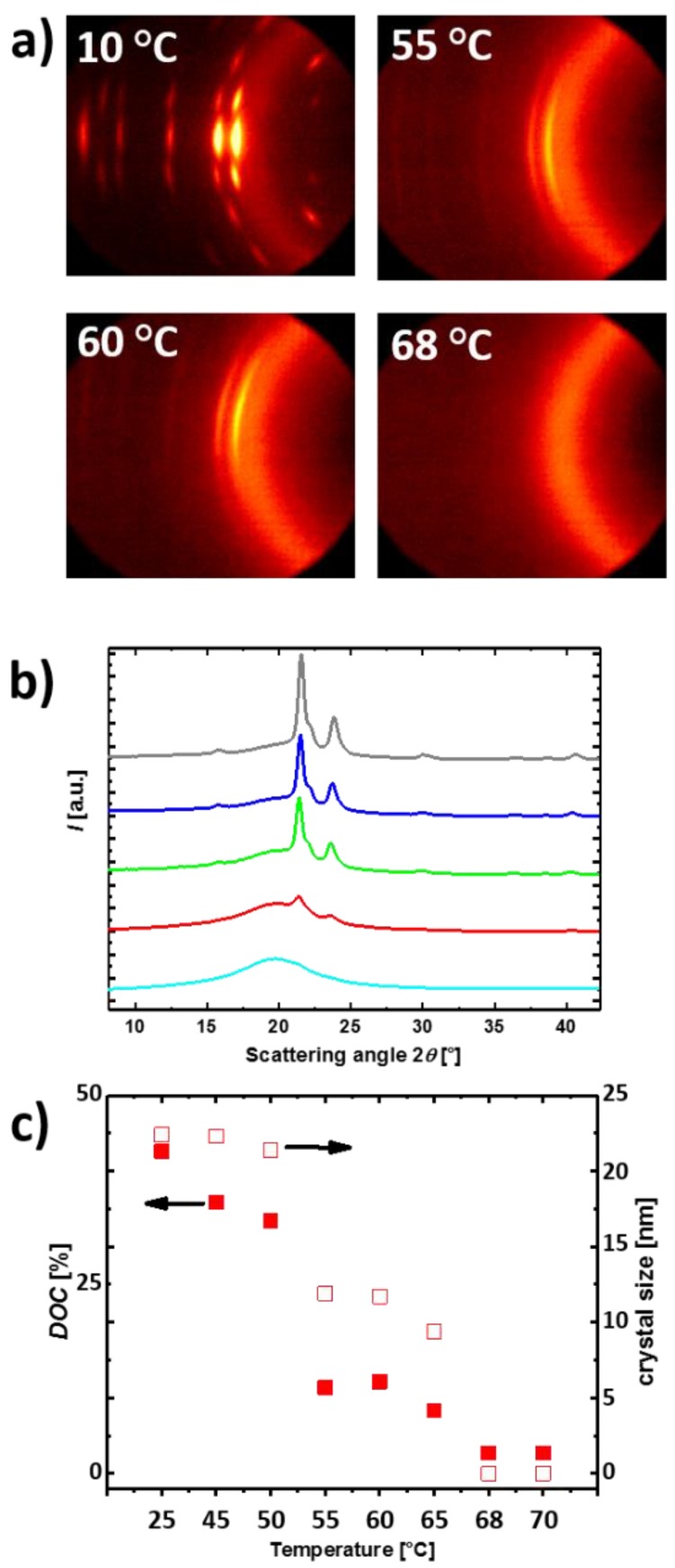
(**a**) Series of 2D WAXS scattering pattern of cPCL*70*-L*30* at different temperatures; (**b**) Scattering intensity curves from WAXS of cPCL*70*-L*30* at 25 (grey line), 50 (blue line), 55 (green line), 60 (red line) and 65 °C (cyan line); (**c**) Temperature dependent evolution of the DOC and crystal size for cPCL*70*-L*30* determined from X-ray scattering.

The WAXS patterns of the programmed cPCL*70*-L*30* during actuation were recorded at various temperatures (10–70 °C) and are shown in Figure 5a. The programmed specimen exhibited highly anisotropic, strong reflections (fiber diagram like) at 10 °C prior to actuation, which is related to highly oriented crystals and a high DOC. As the strongest PCL-(110) reflection was located on the equator, we concluded that the molecular chain axis of the crystals was parallel to the direction of deformation. With increasing temperature, the intensity of the reflections is gradually reduced (Figure 5b) resulting in a reduction of the DOC. This was attributed to the melting of the actuation domain. The degree of anisotropy is also reduced as the spot-like reflections turned into sickle-like reflections, which is related to the large macroscopic contraction of the specimen in the first heating after programming with subsequent loss of crystal orientation to some extent. Nonetheless, at 60 °C some crystallinity remained in the sample and had anisotropic character. This is attributed to the oriented crystalline skeleton in PCL. The apparent average lateral crystal size and DOC of PCL for cPCL*70*-L*30* at different temperatures is shown in Figure 5c. Up to 55 °C, the crystal size was relatively constant, indicating the presence of thermally stable crystals at elevated temperatures. Above 55 °C, rapid melting leads to a decrease in the average crystal size and DOC (Figure 5c), which is indicated by a drop in the values. SAXS patterns recorded at analogue temperatures exhibited highly ordered domains with two-dot diagrams with maxima on the meridian (see Supplementary Figure S6). This indicated a lamellar-like sandwich structure of alternating crystalline/amorphous layers, which were oriented perpendicular to the direction of the deformation. In addition, here the orientation of the skeleton domain was kept at 60 °C, which correlated to the observations from WAXS.

We assume for cPCL*70*-L*30* that the crosslinked polymer network and the linear content undergo a template formation under stretching, in which all polymer chains are oriented preferentially along the stretching direction. This enables oriented recrystallization after partial melting of the actuation domain upon cooling [28]. During actuation, the reversible heating/cooling leads to melting of the linear polymer chains as those are able to form stable crystals, and therefore contribute mostly to the actuation. The linear PCL fraction recrystallizes in the direction of the network structure, which leads to a higher actuation performance in the case where we have the highest amount of linear PCL (30 wt %).

### 3.2. Self-Healing Capabilities

**Figure 6:**
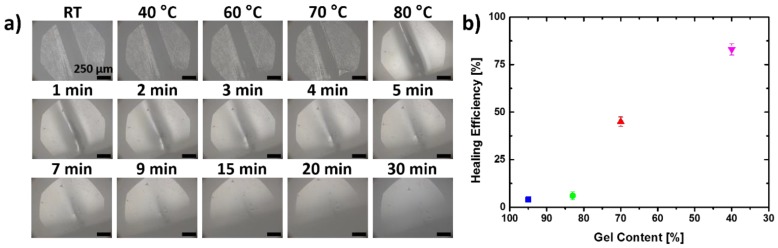
(**a**) Picture series heating a damaged plane film of cPCL*40*-L*60* network at different temperatures and after different time steps leading to closure of the cut due to melting of linear PCL. (spherical shape of image results by the microscopes objective causing this effect); (**b**) Healing efficiency of cPCL*95*-L*05* (blue), cPCL*83*-L*17* (green), cPCL*70*-L*30* (red) and cPCL*40*-L*60* (magenta) vs. gel content estimated by comparison of the mechanical tensile properties of the pristine, damaged and healed sample at ambient temperature.

As an additional function of the extractable fraction of PCL, we expect the ability of self-healing damages in samples for high contents of linear PCL as already described in the literature [11]. But here we would like to obtain a material able to be re-used and reprogrammed after healing. To evaluate suitable candidates, two small damages were applied to the material (width ~250 µm; length ~300 µm; overall width of the specimen: 2 mm; Figure S7) on both sides and heated to different temperatures. A damaged specimen of cPCL*40*-L*60* was heated on a heating stage below an optical microscope and visually inspected for closure of one cut (Figure 6). The respective cut was stable until 60 °C and started to close due to the melting of the extractable mobile PCL chains in the material. Keeping the sample at 80 °C for 30 min leads to a complete closure of the cut for cPCL*40*-L*60* (Figure 6a). The self-healing efficiency for the different materials determined by the comparison of tensile experiments of pristine and healed samples, according to Equation (5), was reduced by decreasing the content of linear polymer in the network (Figure 6b and Figure S8) as also has been shown for other polymer networks [11]. At least 30 wt % of linear polymer is necessary to observe any reasonable changes in the width of the cut.

**Figure 7:**
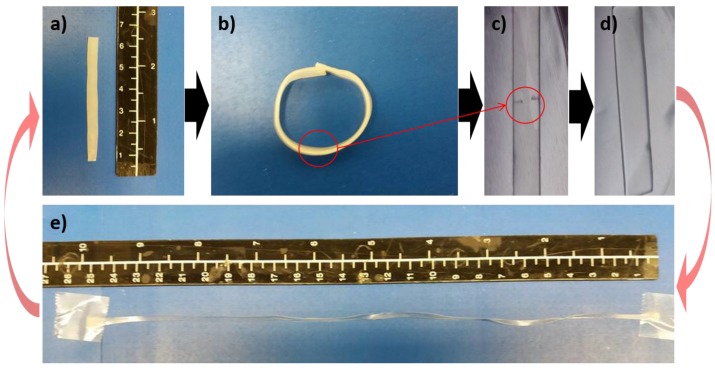
Series of images for cPCL*40*-L*60*: (**a**) pristine specimen; (**b**) bending experiment and (**c**) recovery to the original flat shape by heating above *T*_m_ (healing temperature); (**d**) self-healing capability enables closure of the damages; (**e**) reprogrammability of the sample via stretching to ~250%, and finally recovering to the original shape.

cPCL*40*-L*60* and cPCL*70*-L*30* were used for an additional experiment, which includes a bending experiment, sequential damaging of the specimen via two cuts on the opposite sides, healing, subsequent reprogramming via stretching, and finally recovering to the original shape. In case of cPCL*40*-L*60*, no reversible actuation for bending is observed (as discussed above), but after recovering and cutting, the cut is fully healed at 80 °C (erasing any programmed shape) enabling a sample which can be stretched to at least 250% strain (Figure 7). Heating to 60 °C leads to a recovery of the specimen. cPCL*70*-L*30* showed reversible actuation for a bended sample, but the self-healing efficiency is lowered, and the cuts appear again upon stretching. However, here the stretched sample is able to perform reversible shape-shifts between two states between 60 and 10 °C (Figure S9). 

Therefore, network materials consisting of crosslinked and linear PCL showing stress-free reversible bidirectional shape-memory effect—with a lowered self-healing ability and a material able to show one-way recovery behavior with a high self-healing ability during reprogramming can be generated.

## 4. Conclusions

Multifunctional materials received from randomly crosslinked PCL enabled us to study free-standing bidirectional shape-memory actuation, self-healing capabilities, and reprogrammability of different specimens. Crosslinked PCL thermosets with different amounts of extractable PCL were obtained by different crosslinking techniques (including irradiation and thermal treatment), whereby, interestingly the averaged chain length between two crosslinks and the crosslinking density remained in a similar range. Only the theoretical accessible volume for the reptation of polymer chains, which can be occupied by the linear PCL, was varied, resulting in open or closed compartments in non-continuous structure, which was observed by SEM. Therefore, we assume all thermal or mechanical changes result from the differences in the linear content, which could introduce new functions in the material. All materials showed a reversible bidirectional shape-memory actuation, while for cPCL*70*-L*30* the highest Δε of 24% was determined by stress-free cyclic thermomechanical measurements after stretching. Tensile and X-ray scattering lead to the assumption of templating effects of the network keeping the linear polymer chains oriented in the cyclic heating and cooling experiments. As an additional function, high fractions of linear polymer chains within the network show self-healing capabilities. During the healing step, above the temperatures at which the “memory” of the material is erased, a subsequent programming leads to a new shape. In case of cPCL*40*-L*60*, only a one-way shape-memory effect could be found, while cPCL*70*-L*30* was able to still perform reversible bidirectional actuation.

These findings might be general and could be transferred to other semi-crystalline polymeric materials, thus enabling a broad applicability of this principle. Furthermore, the different crosslinking techniques indicating the formation of similar networks and enabling to control the crosslinked polymer content, could be a good platform for other systems as well. Such shape-memory polymer actuator materials might be capable to show stress-free reversible actuation even after damage allowing technologies for reuse and thereby improving the life cycle of such actuators. Easy processing of such self-healable actuators based on commodity polymer (PCL) make them suitable for readily adaptation for various applications i.e., self-healable artificial muscles for humanoid robotics.

## Figures and Tables

**Table 1:** Summary of physical properties of cPCL networks.

Polymer Network ^(a)^	*T*_m_^(b)^ (°C)	∆*H*_m_^(c)^ (J·g^−1^)	*χ*_c_^(d)^ (%)	*E* ^(e)^ (MPa)	ε_b_^(f)^ (%)	*E* ^(g)^ (MPa)	ε_b_^(h)^ (%)	*G* ^(i)^ (%)
PCL-L*100*	60 ± 1	65 ± 1	48 ± 1	- ^(j)^	- ^(j)^	152 ± 10	790 ± 10	- ^(j)^
cPCL*40*-L*60*	59 ± 1	63 ± 1	47 ± 1	0.06 ± 0.01	1210 ± 20	175 ± 10	910 ± 20	40 ± 2
cPCL*70*-L*30*	59 ± 1	63 ± 1	47 ± 1	1.15 ± 0.1	790 ± 30	210 ± 6	715 ± 35	70 ± 3
cPCL*83*-L*17*	58 ± 1	63 ± 1	47 ± 1	1.61 ± 0.06	310 ± 20	226 ± 13	460 ± 30	85 ± 3
cPCL*95*-L*05*	53 ± 1	61 ± 1	45 ± 1	1.78 ± 0.12	460 ± 15	257 ± 7	550 ± 15	95 ± 1

^(a)^ Polymer network: Named according to the composition of the network of crosslinked and linear contents; ^(b)^ *T*_m_: Melting temperature peak of PCL determined in the second heating curve of DSC measurement; ^(c)^ ∆*H*_m_: Total melting enthalpy calculated as the area under the curve of the melting process; ^(d)^ *χ*_c_: Weight percent crystallinity of PCL calculated from DSC data; ^(e)^ *E*: Young’s modulus determined by tensile tests at 90 °C; ^(f)^ ε_b_: Elongation at break during tensile tests at 90 °C; ^(g)^ *E*: Young’s modulus determined by tensile tests at 25 °C; ^(h)^ ε_b_: Elongation at break during tensile tests at 25 °C; ^(i)^ *G*: Gel contents determined by swelling experiments; ^(**j**)^ non-crosslinked material is completely molten or dissolved under this conditions.

**Table 2:** Summary of physical properties of cPCL networks concerning the crosslinking density and the molecular weight between netpoints.

Polymer Network ^(a)^	*υ*_c_^(b)^ (mol·cm^−3^) (Mooney-Rivlin)	*M*_c_^(c)^ (g·mol^−1^) (Mooney-Rivlin)	*υ*_c_^(d)^ (mol·cm^−3^) (Flory-Rehner)	*M*_c_^(e)^ (g·mol^−1^) (Flory-Rehner)
cPCL*40*-L*60*	1.83·10^−5^	1.71·10^5^	n.d.	n.d.
cPCL*70*-L*30*	1.09·10^−4^	1.05·10^4^	n.d.	n.d.
cPCL*83*-L*17*	2.22·10^−4^	5.14·10^3^	n.d.	n.d.
cPCL*95*-L0*5*	2.32·10^−4^	4.92·10^3^	n.d.	n.d.
cPCL*70*	1.03·10^−4^	1.11·10^4^	1.68·10^−4^	6.77·10^3^
cPCL*83*	1.60·10^−4^	7.11·10^3^	2.39·10^−4^	4.76·10^3^
cPCL*95*	2.14·10^−4^	5.33·10^3^	2.72·10^−4^	4.19·10^3^

^(a)^ Polymer network: Named according to the composition of the network of crosslinked and linear contents; ^(b)^ *υ*_c_: crosslinking density determined according to Mooney Rivlin; ^(c)^ M_c_: average molecular weight between two neighboring netpoints determined according to Mooney-Rivlin; ^(d)^ *υ*_c_: crosslinking density determined according to Flory-Rehner; ^(e)^ *M*_c_: average molecular weight between two neighboring netpoints determined according to Flory-Rehner. n.d.: not determined.

**Table 3:** Summary of reversible actuation properties of cPCL networks.

Polymer Network ^(a)^	*T*_sw,act_^(b)^ (°C)	*T*_sw,rec_^(c)^ (°C)	Δε ^(d)^ (%)
cPCL*40*-L*60*	-	-	3 ± 1
cPCL*70*-L*30*	49 ± 1	58 ± 1	24 ± 1
cPCL*83*-L*17*	47 ± 1	59 ± 1	21 ± 2
cPCL*95*-L*05*	43 ± 1	57 ± 1	20 ± 1

^(a)^ Polymer network: Named according to the composition of the network of crosslinked and linear contents; ^(b)^ *T*_sw,act_: Switching temperatures during actuation (cooling); ^(c)^ *T*_sw,rec_: Switching temperatures during recovery (heating); ^(d)^ Δε: Represents the reversible elongation which is the strain change during actuation.

## Supplementary Materials

The following are available online at http://www.mdpi.com/2073-4360/10/3/255/s1, Figure S1: Cross-sectional SEM micrographs of extracted cPCL which were dried and freeze-fractured (a–c) and networks freeze-dried from dioxane (d–f): (a,d) cPCL95, (b,e) cPCL83, (c,f) cPCL70. Scale bar represents 20 µm. Figure S2: SEM micrographs of the surface of non-extracted (a–c) and extracted networks (d–f): (a,d) cPCL*95*, (b,e) cPCL*83*, (c,f) cPCL*70*. Scale bar represents 20 µm. Figure S3: Comparison of DSC traces during cooling after first heating for: cPCL*95*-L*05* (blue), cPCL*83*-L*17* (green), cPCL*70*-L*30* (red), cPCL*40*-L*60* (magenta), and linear PCL (dashed black line). Figure S4: Comparison of representative stress-strain curves obtained from tensile tests at 25 °C for different crosslinked PCL networks: cPCL*95*-L*05* (blue), cPCL*83*-L*17* (green), cPCL*70*-L*30* (red), and cPCL*40*-L*60* (magenta). Figure S5: Picture series from reversible bidirectional bending experiments after programming in water at 90 °C and cooling to 5 °C. Programmed specimens were placed subsequently in petri dishes with water at 5 °C, 50 °C, and again 5 °C while monitoring the angle changes. Figure S6: Changes of the scattering pattern determined by 2D SAXS during the bidirectional actuation of programmed cPCL*70*-L*30* at different temperatures: 10 °C, 50 °C, 55 °C, and 60 °C. Figure S7: Schematic representation of crosslinked PCL networks (blue lines) containing linear non-crosslinked PCL (green), which could be damaged by a cut leading to a destruction of the situation, while the mobile fraction of PCL is able to close the damage upon heating to temperatures above T_m_. Figure S8: Comparison of tensile curves obtained for cPCL*40*-L*60* (a,b) and cPCL*70*-L*30* (c,d) for damaged samples (triangular symbols; enlarged in b and d of the respective material), pristine samples (square symbols) and healed samples (spherical symbols) in a and c. Figure S9: Series of images for cPCL*70*-L*30*: (a) pristine specimen; (b) bending experiment and reversible actuation; (c) damaging via two cuts on the opposite sides; (d) self-healing capability enables closure of the damages; (e) reprogrammability of the sample via stretching to ~350% (showing reversible actuation), and finally recovering to the original shape. Table S1: Swelling degree Q extracted cPCL networks.
