# Supplementary material for: Extractable Free Polymer Chains Enhance Actuation Performance of Crystallizable Poly(ε-caprolactone) Networks and Enable Self-Healing
Source: Polymers (Basel). 2018 Mar 1;10(3):255. doi: 10.3390/polym10030255 (PMC6415158; doi:10.3390/polym10030255)
Supplement: Supplementary file 1 [file polymers-10-00255-s001.pdf]

Supporting Materials: Extractable Free Polymer Chains Enhance Actuation Performance of Crystallizable Poly(ε-caprolactone) Networks and Enable Self-Healing

Muhammad Farhan[1,2], Tobias Rudolph[1], Ulrich Nöchel[1], Karl Kratz[1], Andreas Lendlein[1,2*]

[1] *Institute of Biomaterial Science and Berlin-Brandenburg Center for Regenerative Therapies (BCRT), Helmholtz-Zentrum Geesthacht, Kantstr. 55, 14513 Teltow, Germany;* muhammad.farhan@hzg.de, tobias.rudolph@hzg.de, ulrich.noechel@hzg.de, karl.kratz@hzg.de, andreas.lendlein@hzg.de

[2] *Institute of Chemistry, University of Potsdam, 14476 Potsdam-Golm, Germany*; muhammad.farhan@hzg.de, andreas.lendlein@uni-potsdam.de

* Correspondence: andreas.lendlein@hzg.de; Tel.: +49-3328-352 235

Throughout the supporting material section networks are either called cPCLXX-LYY or cPCLXX, where the numbers indicate the weight fraction of crosslinked PCL (cPCL) and linear content (L). cPCLXX are intensively extracted networks no longer containing any extractable linear PCL macromolecules.

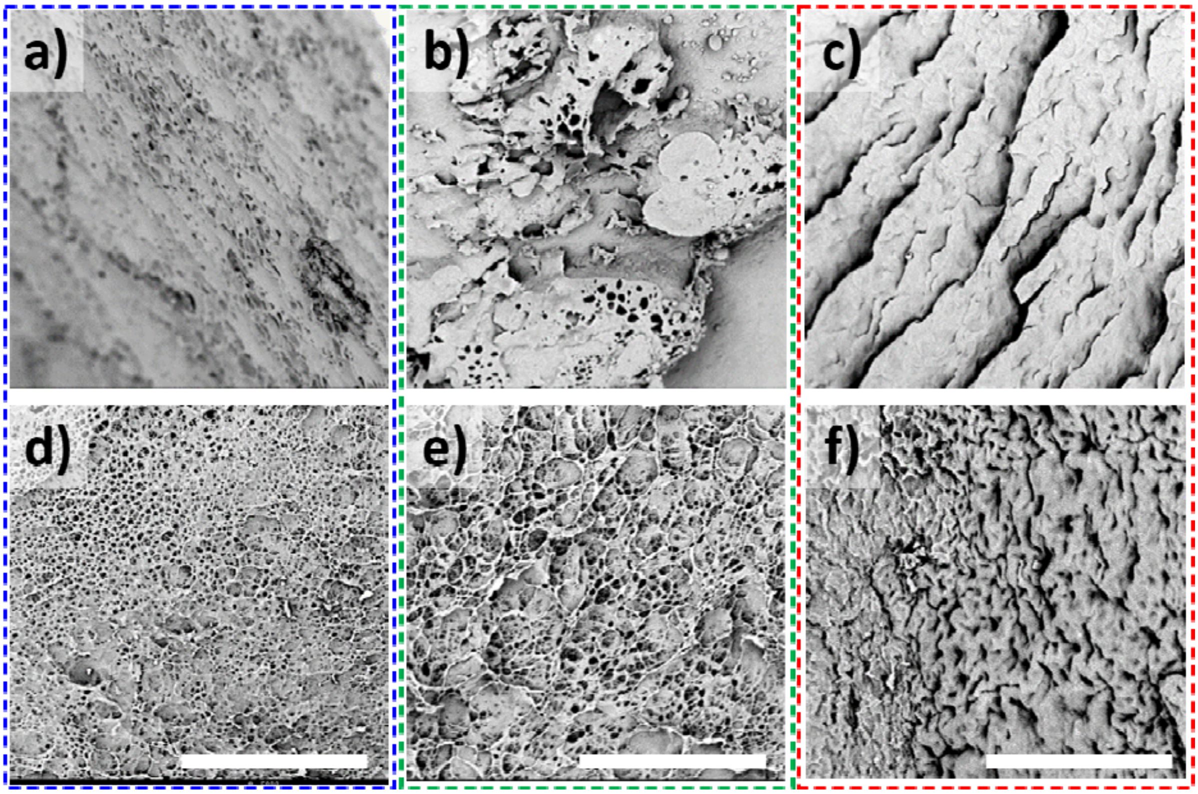

Figure S1. Cross-sectional SEM micrographs of extracted cPCL which were dried and freeze-fractured **(a,b,c)** and networks freeze-dried from dioxane **(d,e,f)**: **(a,d)** cPCL*95*, **(b,e)** cPCL*83*, **(c,f)** cPCL*70*. Scale bar represents 20 μm.

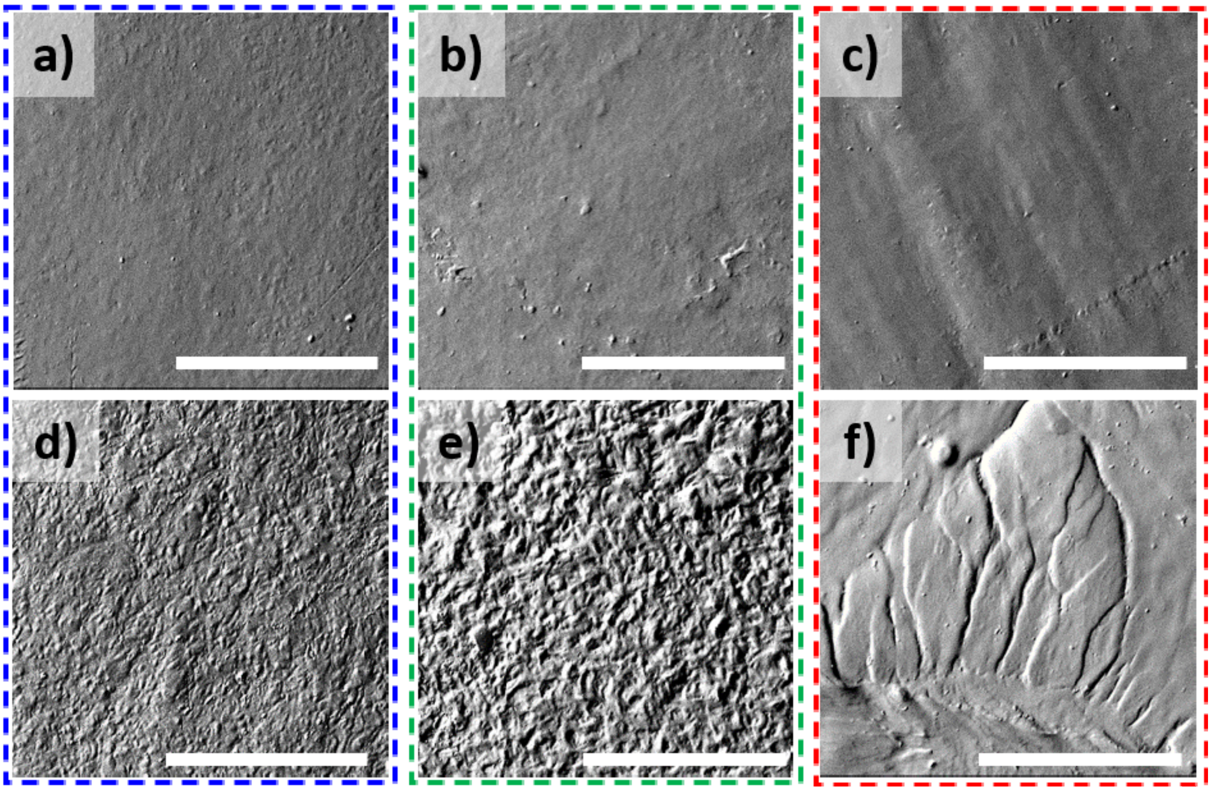

Figure S2. SEM micrographs of the surface of non-extracted **(a,b,c)** and extracted networks **(d,e,f)**: **(a,d)** cPCL*95*, **(b,e)** cPCL*83*, **(c,f)** cPCL*70*. Scale bar represents 20 µm.

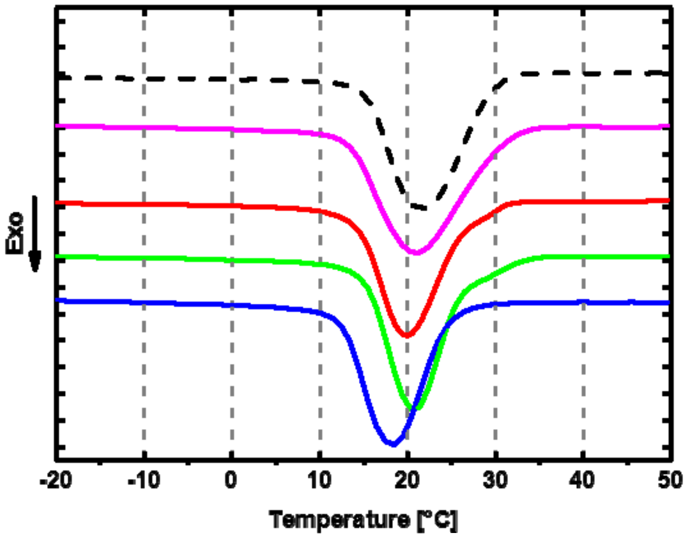

Figure S3. Comparison of DSC traces during cooling after first heating for: cPCL*95*-L*05* (blue), cPCL*83*-L*17* (green), cPCL*70*-L*30* (red), cPCL*40*-L*60* (magenta), and linear PCL (dashed black line).

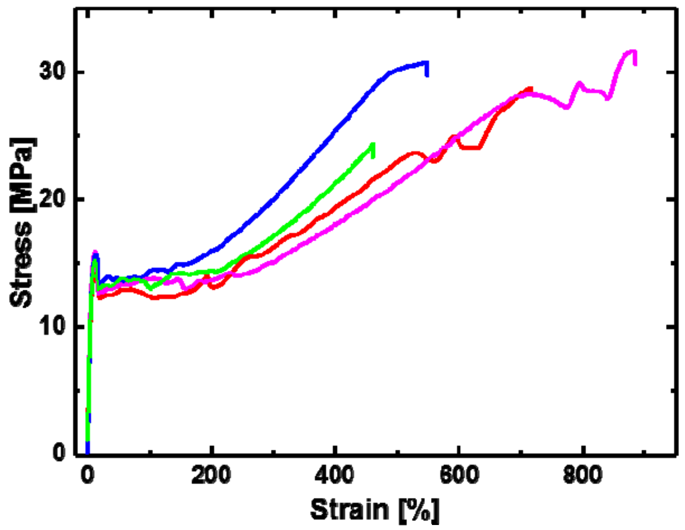

Figure S4. Comparison of representative stress-strain curves obtained from tensile tests at 25 °C for different crosslinked PCL networks: cPCL*95*-L*05* (blue), cPCL*83*-L*17* (green), cPCL*70*-L*30* (red), and cPCL*40*-L*60* (magenta).

Table S1. Swelling degree Q for extracted cPCL networks.

Polymer network [a)]	Q [b)] (%)
cPCL*70*-L*30*	3150 ± 50
cPCL*83*-L*17*	2005 ± 20
cPCL*95*-L*05*	1445 ± 25

a) Polymer network: Named according to the composition of the network of crosslinked and linear contents; **b)** Q: Swelling degree.

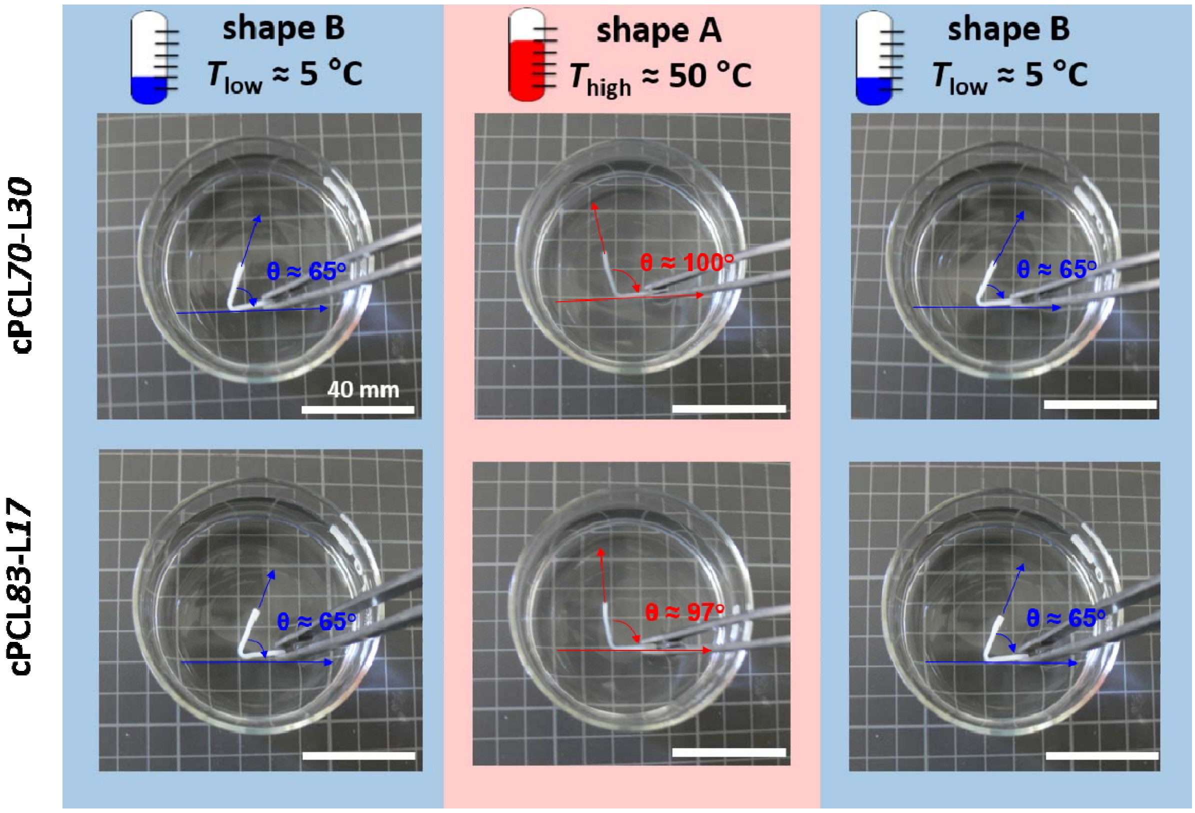

Figure S5. Picture series from reversible bidirectional bending experiments after programming in water at 90 °C and cooling to 5 °C. Programmed specimens were placed subsequently in petri dishes with water at 5 °C, 50 °C, and again 5 °C while monitoring the angle changes.

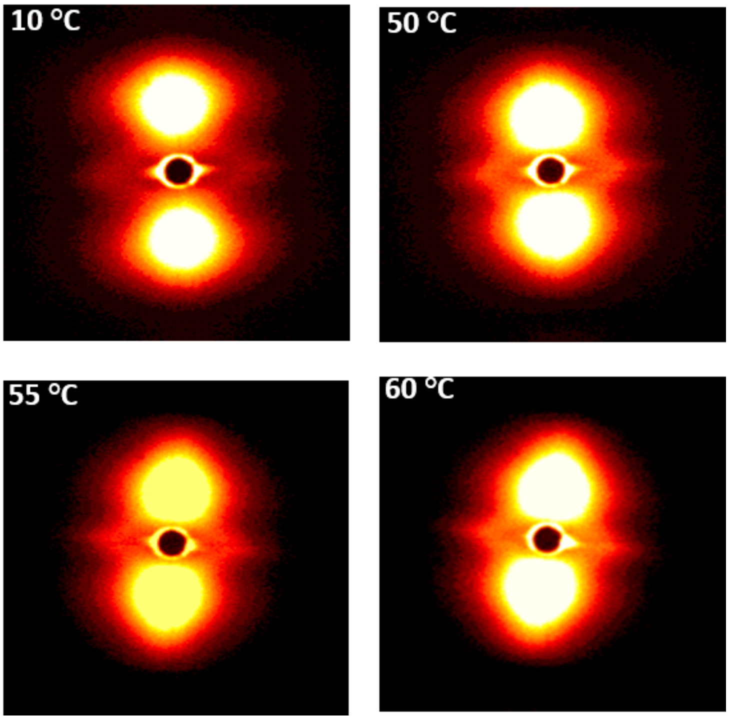

Figure S6. Changes of the scattering pattern determined by 2D SAXS during the bidirectional actuation of programmed cPCL*70*-L*30* at different temperatures: 10 °C, , 50 °C, 55 °C, and 60 °C.

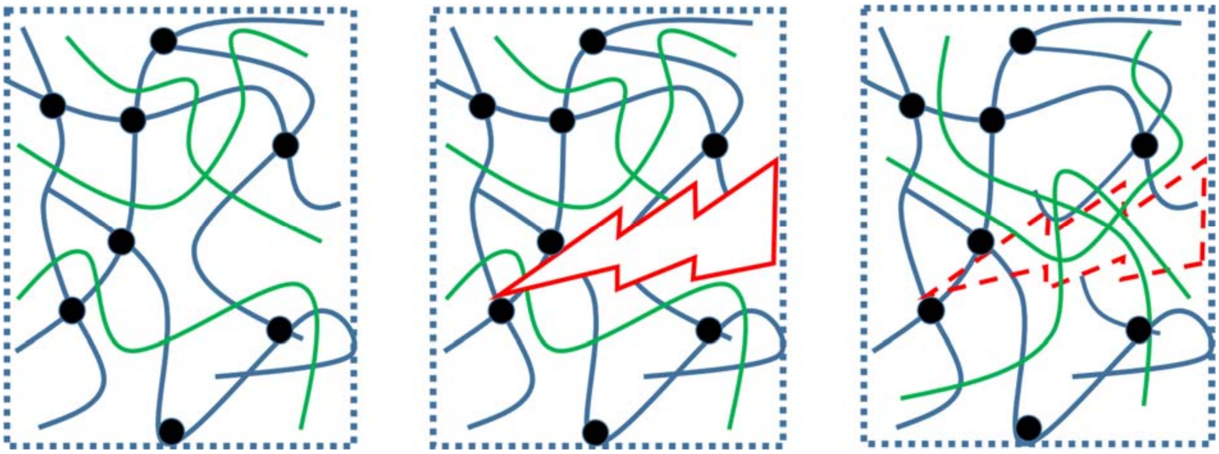

Figure S7. Schematic representation of crosslinked PCL networks (blue lines) containing linear non-crosslinked PCL (green), which could be damaged by a cut leading to a local destruction of the covalently crosslinked network, while the mobile fraction of PCL is able to close the damage upon heating to temperatures above T_m.

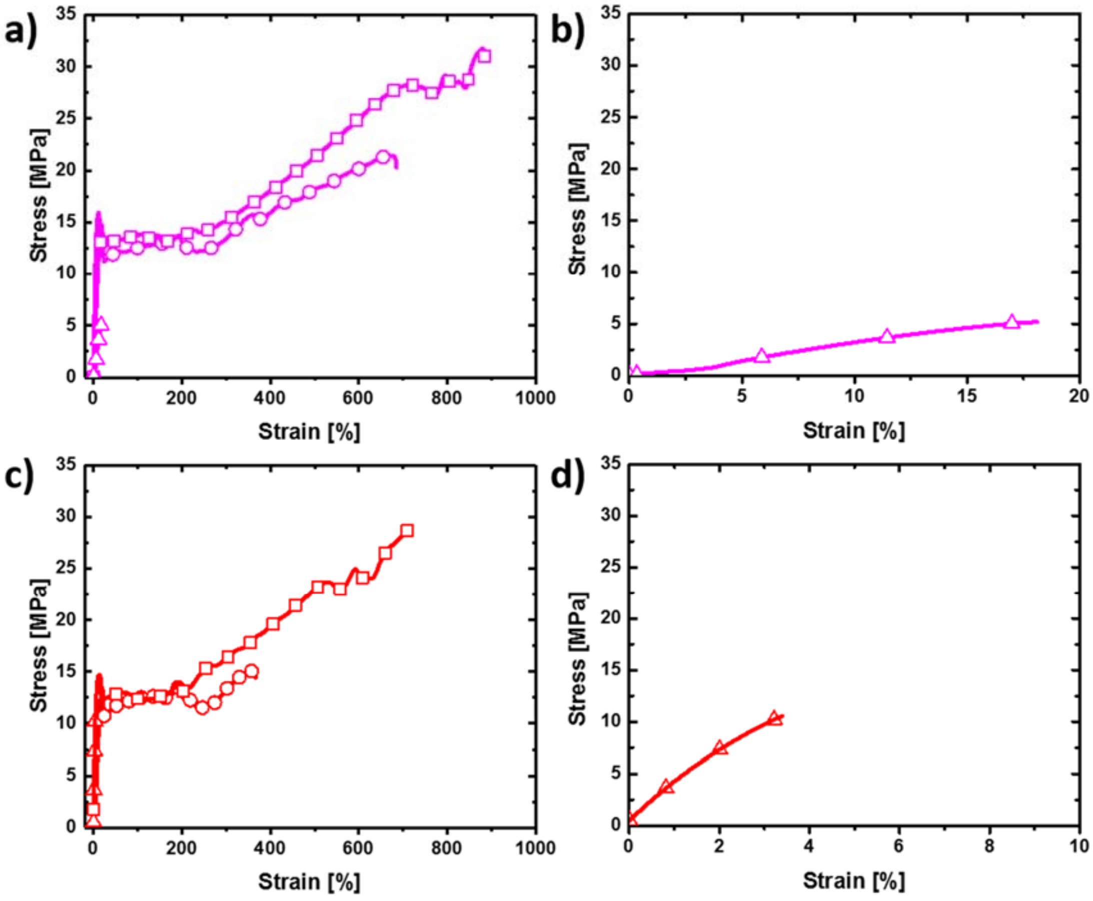

Figure S8. Comparison of tensile curves obtained for cPCL*40*-L*60* **(a,b)** and cPCL*70*-L*30* **(c,d)** for damaged samples (triangular symbols; enlarged in b and d of the respective material), pristine samples (square symbols) and healed samples (spherical symbols) in a and c.

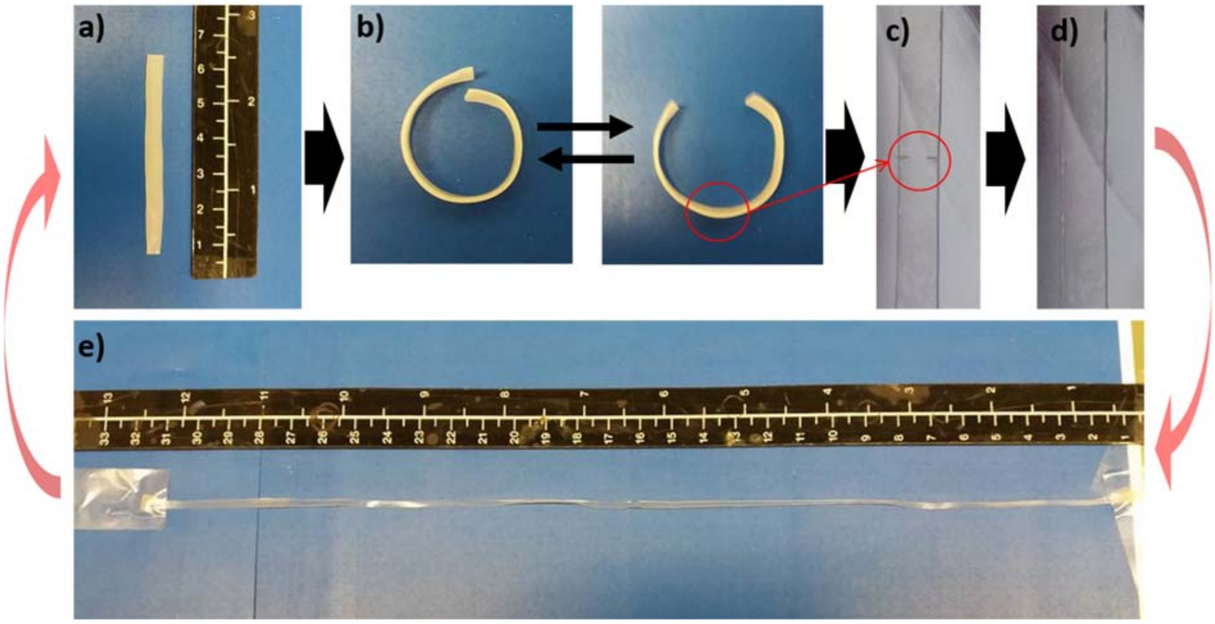

Figure S9. Series of images for cPCL*70*-L*30*: **a)** pristine specimen; **b)** bending experiment and reversible actuation; **c)** damaging via two cuts on the opposite sides; **d)** self-healing capability enables closure of the damages; **e)** reprogrammability of the sample via stretching to ~350% (showing reversible actuation), and finally recovering to the original shape.
